# Motor unit number index in Bell's palsy: A potential electrophysiological biomarker for early evaluation

**DOI:** 10.1002/brb3.3632

**Published:** 2024-09-15

**Authors:** Mengjie Chen, Mingxia Zhu, Xiuli Li, Jingtao Pi, Xinhong Feng

**Affiliations:** ^1^ Department of Neurology, Beijing Tsinghua Changgung Hospital, School of Clinical Medicine Tsinghua University Beijing China; ^2^ Department of Neurology and Neurological Rehabilitation, Shanghai Yangzhi Rehabilitation Hospital (Shanghai Sunshine Rehabilitation Center), School of Medicine Tongji University Shanghai China

**Keywords:** Bell's palsy, early evaluation, MUNIX

## Abstract

**Introduction:**

Reliable, noninvasive early diagnostics of neuromuscular function in Bell's palsy, which causes facial paralysis and reduced quality of life, remain to be established. Here, we aimed to evaluate the utility of the motor unit number index (MUNIX) for the quantitative electrophysiological assessment of early‐stage Bell's palsy, its correlation with clinical assessments, changes following treatment, and association with clinical prognosis.

**Methods:**

MUNIX measures were recorded from the bilateral zygomaticus, orbicularis oculi, and orbicularis oris muscles of 10 healthy individuals and 64 patients with Bell's palsy. The patients were assessed by two specialist neurologists using the House–Brackmann and Sunnybrook Facial Grading Systems. Repeat assessments were performed on 20 patients with Bell's palsy who received treatment. Additionally, the 64 patients were reassessed using clinical scales after a 1‐month interval.

**Results:**

The MUNIX values of the main affected muscles on the affected side were lower than those on the healthy side in patients with Bell's palsy (*p* < .05). The MUNIX measurements significantly correlated with the clinical facial nerve palsy scale scores (*p* < .05). Significant improvements were observed in the MUNIX values on repeat testing following treatment (*p* < .05). The baseline motor unit size index (the compound muscle action potential amplitude divided by MUNIX) was positively associated with improved clinical presentation after 1 month (*p* < .05).

**Conclusion:**

MUNIX can be used as an electrophysiological biomarker for the quantitative assessment of facial nerve palsy and treatment response, and as a prognostic biomarker, in patients with early Bell's palsy, and is recommended as a complement to conventional neurophysiological examinations.

## Graphical abstract

Motor unit number index (MUNIX) in Bell's palsy: A potential electrophysiological biomarker for early evaluation

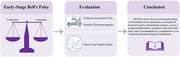

## 1 INTRODUCTION

Bell's palsy is a clinically common form of acute peripheral facial nerve palsy that occurs at all ages (Eviston et al., 2015), with an annual incidence rate of 11.5 to 53 cases per 100,000 individuals (Baugh et al., 2013a). Most symptoms of facial muscle paralysis are unilateral, and most patients begin to show improvement 2−3 weeks after symptom onset. However, 30% of patients do not ultimately achieve full recovery. Asymmetry in facial form and movement caused by Bell's palsy affects patient appearance as well as daily living behaviors including eating, speech, and communication, and common complications cause considerable physical and psychological distress (Tiemstra & Khatkhate, 2007).

In the early stages of the disease, the facial nerve becomes edematous, demyelinated, and degenerative (George et al., 2020). Current electrophysiological tests to objectively assess facial neuromuscular function include electroneurography (EnoG), blink reflex (BR), and needle electromyography (nEMG) (Guntinas‐Lichius et al., 2020; Urban et al., 2020). EnoG and BR measure the conduction properties of the facial nerve and integrity of the facial nerve pathway (Kennelly, 2019). nEMG is the standard method for accurately diagnosing facial nerve damage; however, nEMG has a low positivity rate in the first week of the disease and is, therefore, performed at least 6 days after onset. Additionally, nEMG is a minimally invasive technique that causes pain and discomfort to patients, and is not suitable for long‐term continuous dynamic testing of facial nerve function (Granger, 1976; Guntinas‐Lichius et al., 2020). In turn, ENoG can be used to quantitatively assess the degree of facial nerve degeneration and predict recovery soon after disease onset. However, achieving consensus regarding the site and timing of ENoG measurement remains elusive owing to discrepancies in the results. This can be attributed to the variance in cutoff values for ENoG set by previous studies, differences in the length of follow‐up periods, and the relatively small number of studies available (Remenschneider et al., 2017). In addition, the prognosis of Bell's palsy includes not only complete recovery of facial nerve function but also the presence of complications such as facial spasms and joint band movements. Therefore, ENoG has limitations in terms of evaluating lesion progression and guiding treatment (Azuma et al., 2020, 2018). The standard treatment for Bell's palsy is oral steroid therapy, which is widely used in clinical practice. However, methods to objectively assess the efficacy of treatment and follow‐up with regard to neuromuscular function are lacking (Heckmann et al., 2019). The House–Brackmann Grading System (HBGS) and Sunnybrook Facial Grading System (SFGS) have been widely used as relatively simple and easy clinical assessment tools in patients with Bell's palsy (House & Brackmann, 1985; Ross et al., 1994).

The motor unit number index (MUNIX) is an electromyographic technique developed by Nandedkar et al. in 2004 that provides an index of the number of motor units in muscle (Nandedkar et al., 2004). For a given muscle, a directly related index, the motor unit size index (MUSIX), requires the compound muscle action potential (CMAP) and progressive grade of voluntary muscle contraction, recorded via surface electromyography as a surface interference pattern (SIP) of muscle contraction. MUSIX is calculated by dividing the CMAP amplitude by MUNIX. The use of CMAP and SIP provides the number of motor units in an ideal situation, which corresponds to the MUNIX number in a given SIP region and has the advantages of being easy, noninvasive, and reproducible. However, the value of MUNIX in assessing facial nerve function in the early stages of Bell's palsy and its relationship with disease severity remain unclear.

Therefore, this study aimed to explore (1) whether MUNIX demonstrates early sensitivity in the diagnosis of Bell's palsy by comparing the MUNIX values of the affected and healthy sides in patients with early Bell's palsy; (2) the correlation between MUNIX values and clinical scores (HBGS and SFGS) to determine the clinical relevance of MUNIX in Bell's palsy diagnosis; (3) changes in MUNIX values after receiving standardized therapy and determine the value of MUNIX during follow‐up; and (4) relationship between baseline MUSIX levels and clinical prognosis. Together, this information will provide effective information regarding the use of MUNIX for clinical decision‐making as an early diagnostic aid in Bell's palsy, enabling graded management and timely intervention in patients with potentially poor prognosis, and reducing adverse outcomes, thereby affording individualized and precise treatment of patients with Bell's palsy.

## 2 METHODS

### 2.1 Participants

A total of 64 patients with Bell's palsy diagnosed within 7 days of onset were included in the present study. All participants with Bell's palsy were prospectively recruited at Beijing Tsinghua Changgung Hospital from August 2021 to August 2023. In addition, 10 healthy individuals (no history of Bell's palsy or other disorders that cause facial muscle dysfunction) underwent MUNIX studies performed at 1‐month intervals to verify the reproducibility of the index. Twenty of the 64 enrolled patients with Bell's palsy who received standardized treatment were followed up at 1‐month intervals to assess MUNIX and clinical scale (HBGS and SFGS) values. The 64 recruited patients were reassessed using the clinical scales after a 1‐month interval of standardized treatment. Notably, to avoid individual differences in facial structure and coordination, and to consider the characteristics of unilateral onset of Bell's palsy, the healthy side was utilized as the reference value for each patient, rather than matched healthy controls. The study protocol was approved by the Human Ethics Committees (Beijing Tsinghua Changgung Hospital, School of Clinical Medicine, Tsinghua University, China). All participants gave informed consent.

Inclusion criteria were as follows: (1) Patients aged ≥18 years, who met the diagnostic criteria of Bell's palsy, exhibited acute onset and unilateral peripheral facial paralysis, and had available results of a detailed neurological exam with specific emphasis on cranial nerve V (facial nerve) and motor impairments (e.g., inability to raise eyebrows, close eyes and maintain closure upon clinician attempts to open them, puff out cheeks, and show teeth) (Baugh et al., 2013b); (2) onset time within 7 days, and no treatment was received; and (3) other causes of unilateral motor deficits ruled out by other investigations. Patients with multiple cranial neuritis caused by, for example, traumatic, central, tumor, or other secondary facial paralysis, or viral infection were excluded.

### 2.2 Testing methods

#### 2.2.1 MUNIX

The MUNIX reported by Nandedkar et al. was applied to healthy individuals and patients with Bell's palsy (Nandedkar et al., 2010, 2018). MUNIX measurements were performed using an American Nicolet six‐channel EMG‐evoked potential instrument, Additional MUNIX testing software was installed on the EDX EMG system to facilitate high‐quality data collection at different levels of voluntary contraction. Stimulation conditions for maximum CMAP were as follows: pulse width, 0.2 ms; diameter and spacing between the anode and cathode of the stimulator probe, 2 cm.

**FIGURE 1:**
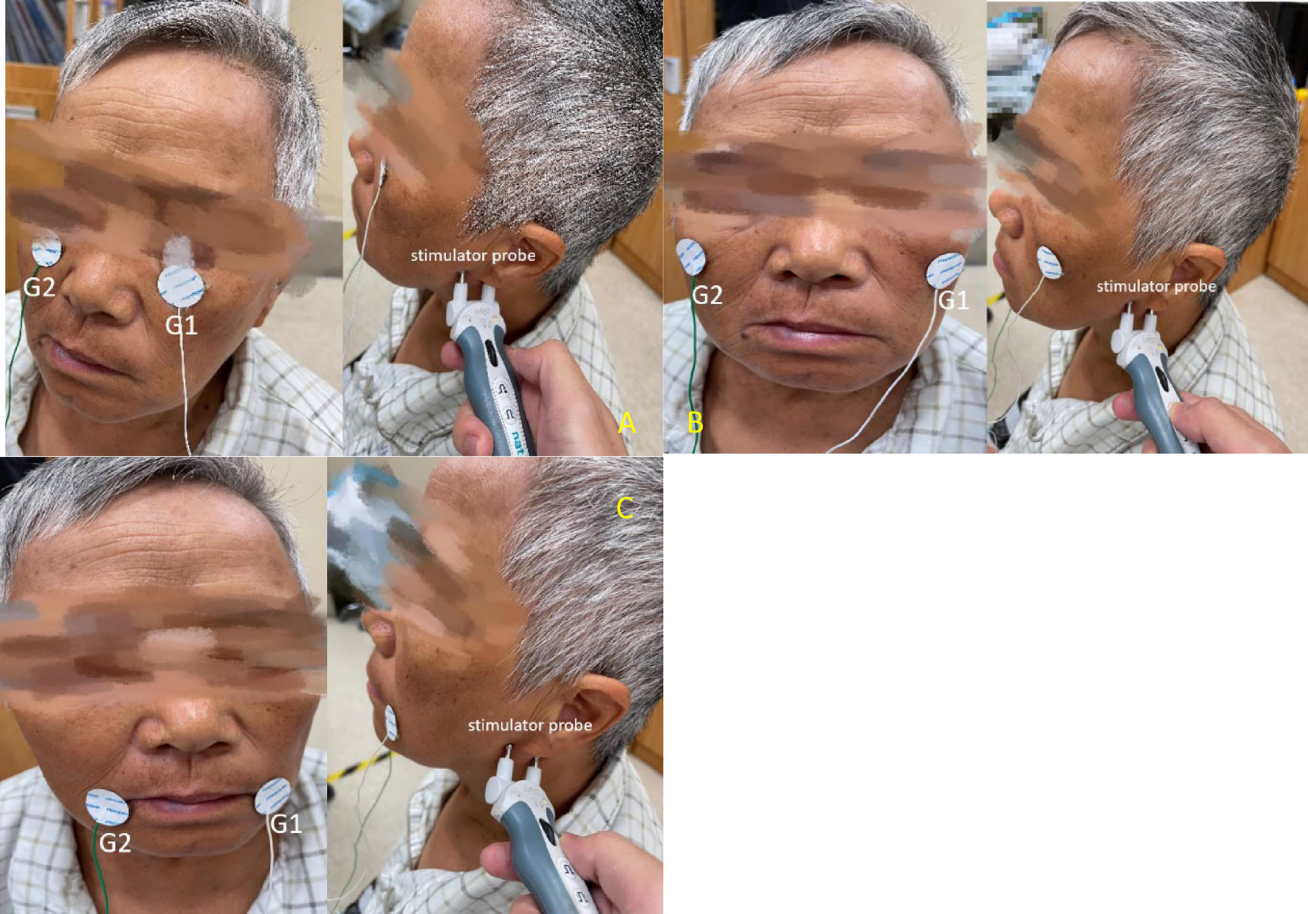
a) The placement of the EMG G1/G2 electrodes on the orbicularis oculi muscle. (b) The placement of the EMG G1/G2 electrodes on the zygomatic muscle. (c) The placement of the EMG G1/G2 electrodes on the orbicularis oris muscle.

Before testing, the face temperature of each participant was checked and controlled to 32°C. The greatest stimulation of the facial nerve was applied in the anterior tragus, in front of the lower ear. The maximal CMAP was recorded bilaterally from the orbicularis oculi, zygomatic, and orbicularis oris muscles, adjusting the position of the active electrode (i.e., G1 was placed on the affected side of the corresponding facial muscle and G2 on the healthy side) (Figure 1). Informed consent was obtained from the patient following the demonstration using an electrode position placement chart.

**FIGURE 2:**
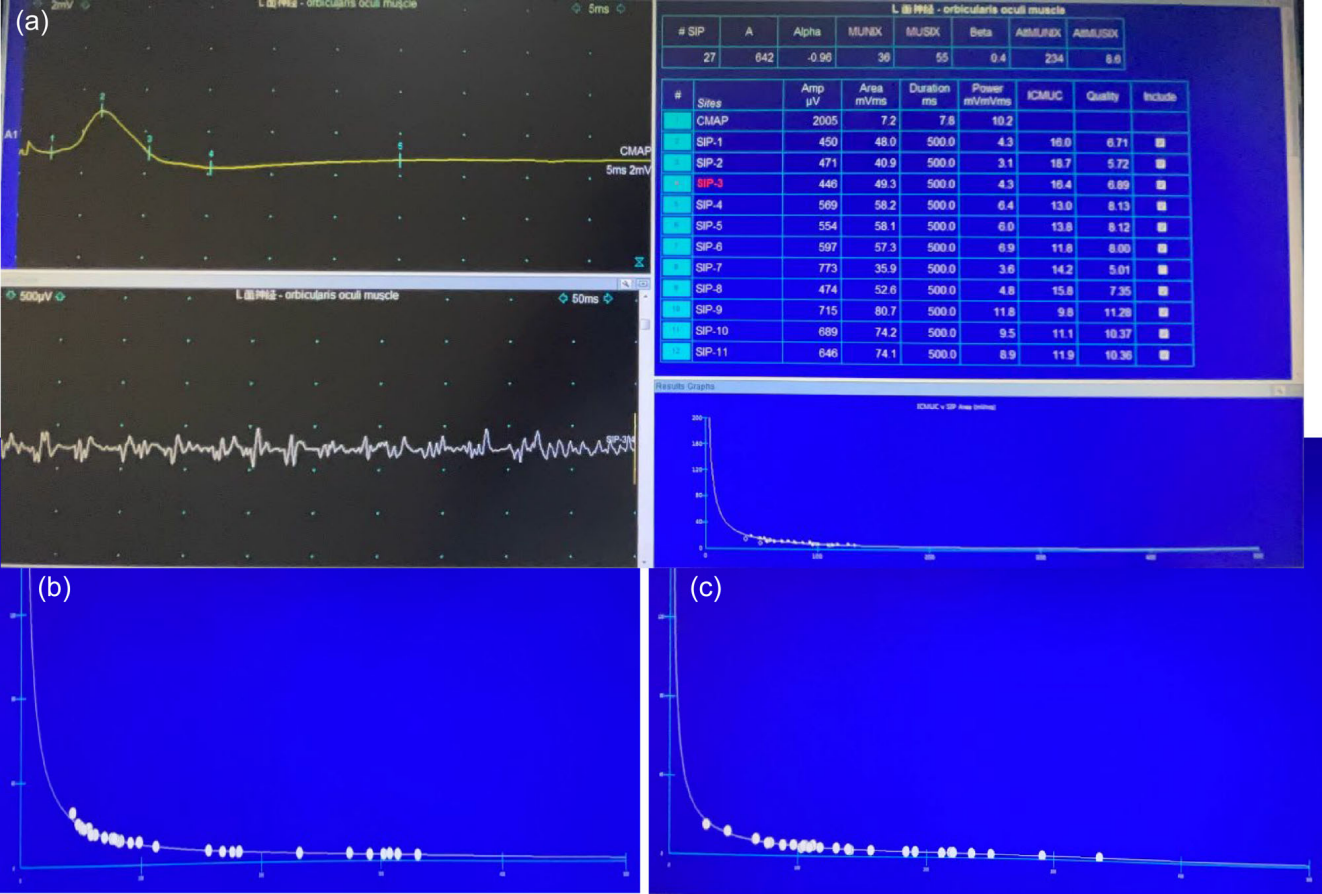
(a) MUNIX operating page. (b) ICMUC v SIP Area (mV x ms) of unaffected side on the zygomatic muscle (ICMUC is the vertical coordinate and the distance of each frame is 40; SIP Area is the horizontal coordinate and the distance of each frame is 100). (c) ICMUC v SIP Area (mV x ms) of affected side on the zygomatic muscle. (ICMUC is the vertical coordinate and the distance of each frame is 40; SIP Area is the horizontal coordinate and the distance of each frame is 100).

The MUNIX calculations were not performed when the CMAP amplitude was < 0.5 mV. The SIP was recorded under five different forces during the muscle contractions of the tested participants (10% or slight, 25%, 50%, submaximal, and maximal contraction). At least 20 SIP recordings were obtained. The orbicularis oculi, zygomatic, and orbicularis oris muscles were activated by eye closing, mouth grinning, and mouth pouting, respectively. The data were exported to an Excel file and used to calculate the MUNIX and MUSIX values (automated analysis is shown in Figure 2). MUSIX was calculated by dividing the CMAP amplitude by MUNIX. The MUNIX sum value was obtained by adding the total MUNIX values of all the tested muscles. The MUSIX sum value was obtained by adding the total MUSIX values of all the tested muscles.

#### 2.2.2 Clinical assessment

All 64 patients with Bell's palsy underwent HBGS and SFGS testing. The HBGS grading scale is divided into six levels, I–VI, with higher levels indicating more severe facial nerve palsy (Yen et al., 2003). The SFGS has three components: static symmetry, voluntary motor symmetry, and joint band movement, each of which has its own observations and scoring criteria, with 0 indicating complete paralysis, and 100 reflecting normal function (Neely et al., 2010). To avoid experimental errors, the HBGS and SFGS were scored by two experienced neurologists.

### 2.3 Statistical methods

All measurements were performed using IBM SPSS (version 24).

The distribution of all variables was tested by the one‐sample Kolmogorov−Smirnov test (sample size > 50) and Shapiro−Wilk test (sample size ≤50).

The test‐retest reliability of MUNIX in healthy controls was tested by the intraclass correlation coefficient (ICC) by using a 2‐way, mixed effects model looking for absolute agreement (Koo & Li, 2016). ICC values close to 1.0 indicate a high degree of similarity between the results and ICC > 0.75 indicates good reliability of the results.

For differences between the affected and healthy side of same person, and changes in variables (MUNIX values of Bell's palsy patients before and after treatment) across follow‐up assessments: the parametric variables were measured using a paired, two‐tailed Student's *t*‐test, and nonparametric variables were assessed using the Wilcoxon signed ranks test.

Receiver operating characteristic (ROC) curve analysis was used for diagnostic test accuracy studies.

Spearman's correlation and Pearson's correlation coefficient were applied to evaluate the statistical correlation between the variables. (GraphPad Prism 8.0.1).

*p*‐Values < .05 were considered significant.

## 3 RESULTS

### 3.1 Demographics

Sixty‐four patients (32 female; age range 20–78 years; mean age 42.03 years) were included, all patients had untreated Bell's palsy within 7 days of onset. Ten healthy controls (5 female; age range 23–65 years; mean age 28.6 years) were included for the repeat MUNIX study.

### 3.2 MUNIX and MUSIX values

First, repeat MUNIX was tested in controls, measuring intervals at 1‐month intervals. ICC was 0.996 for MUNIX sum values, which is higher than MUNIX of a single facial muscle. ICC was 0.995 for orbicularis oculi muscle, then zygomatic muscle (0.988), and orbicularis oris muscle (0.970). One‐sample Kolmogorov−Smirnov test was used for MUNIX and MUSIX, parametric variables: MUNIX orbicularis oculi muscle (affected side), MUNIX orbicularis oris muscle (healthy side), MUNIX SUM (healthy side). All other data belong to nonparametric variables, so we chose the Wilcoxon signed ranks test for assessment (Table 1). MUNIX values were significantly higher in controls (healthy side) than the affected side of Bell's palsy and MUSIX values were significantly lower in the control group.

**TABLE 1:** MUNIX and MUSIX values in healthy side and affected side of patients with Bell's palsy.

Healthy side‐affected side (*n* = 64) ^a^	Z	*p*‐values
MUNIX orbicularis oculi muscle	−6.835^b^	.000
MUSIX orbicularis oculi muscle	−4.533^c^	.000
MUNIX zygomatic muscle	−6.659^b^	.000
MUSIX zygomatic muscle	−4.077^c^	.000
MUNIX orbicularis oris muscle	−6.686^b^	.000
MUSIX orbicularis oris muscle	−3.860^c^	.000
SUM MUNIX	−6.955^b^	.000
SUM MUSIX	−5.036^c^	.000

*Note*: Nonparametric variables were assessed using Wilcoxon signed ranks test.

Abbreviations: MUNIX, motor unit number index; MUSIX, motor unit size index.

^a^Wilcoxon signed ranks test.

^b^Based on negative ranks.

^c^Based on positive ranks.

### 3.3 ROC curve analysis was used for diagnostic test accuracy studies

**FIGURE 3:**
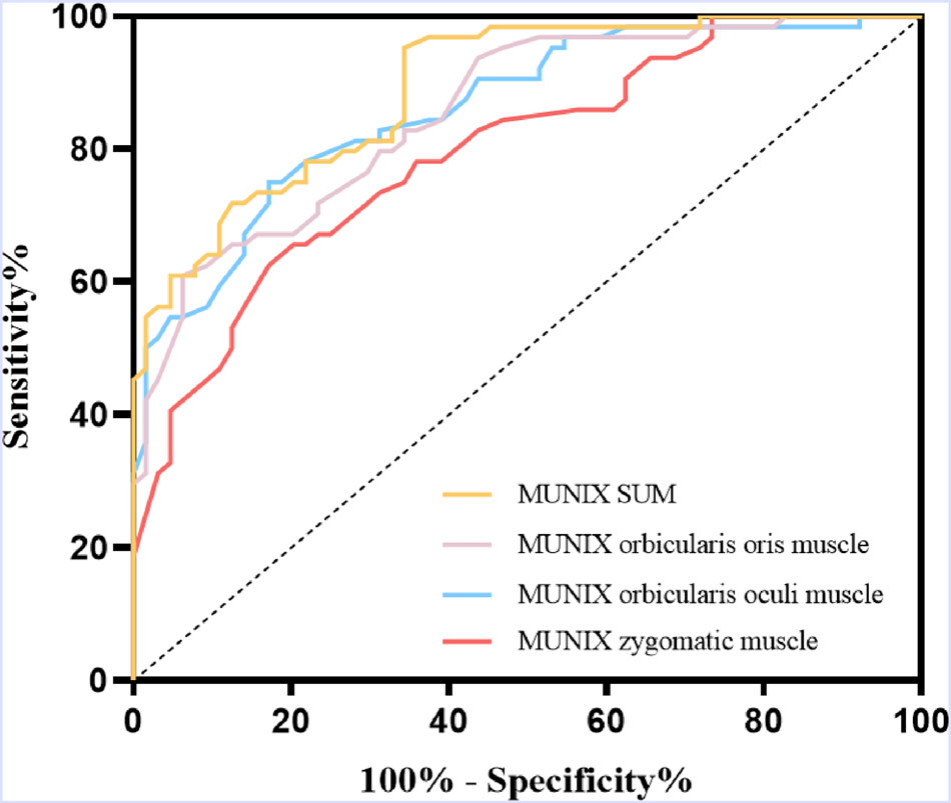
ROC curve analysis was used for diagnostic test accuracy studies.

We used the preferred approach for diagnostic test accuracy studies—ROC curve analysis to analyze the MUNIX values of three muscles and MUNIX SUM in Figure 3.

MUNIX SUM:AUC(Area Under the Curve of ROC) = 0.892; 95%CI 0.840～0.945; *p* = .000; max Youden index = 0.609, CUTOFF = 116.5, sensitivity = 95.3%, specificity = 65.6%, positive predictive value = 73.5%, negative predictive value = 93.3%.

MUNIX orbicularis oris muscle: AUC = 0.856; 95%CI 0.793～0.918; *p* = .000; max Youden index = 0.546, CUTOFF = 19.5, sensitivity = 60.9%, specificity = 93.7%, positive predictive value = 90.7%, negative predictive value = 70.6%.

MUNIX orbicularis oculi muscle: AUC = 0.861; 95%CI 0.799～0.923; *p* = .000; max Youden index = 0.578, CUTOFF = 27.5, sensitivity = 75%, specificity = 82.8%, positive predictive value = 81.4%, negative predictive value = 76.8%.

MUNIX zygomatic muscle: AUC = 0.795; 95%CI 0.720～0.871; *p* = .000; max Youden index = 0.453, CUTOFF = 25.5, sensitivity = 62.5%, specificity = 82.8%, positive predictive value = 78.4%, negative predictive value = 68.8%.

### 3.4 Correlations between MUNIX and clinical assessments

**FIGURE 4:**
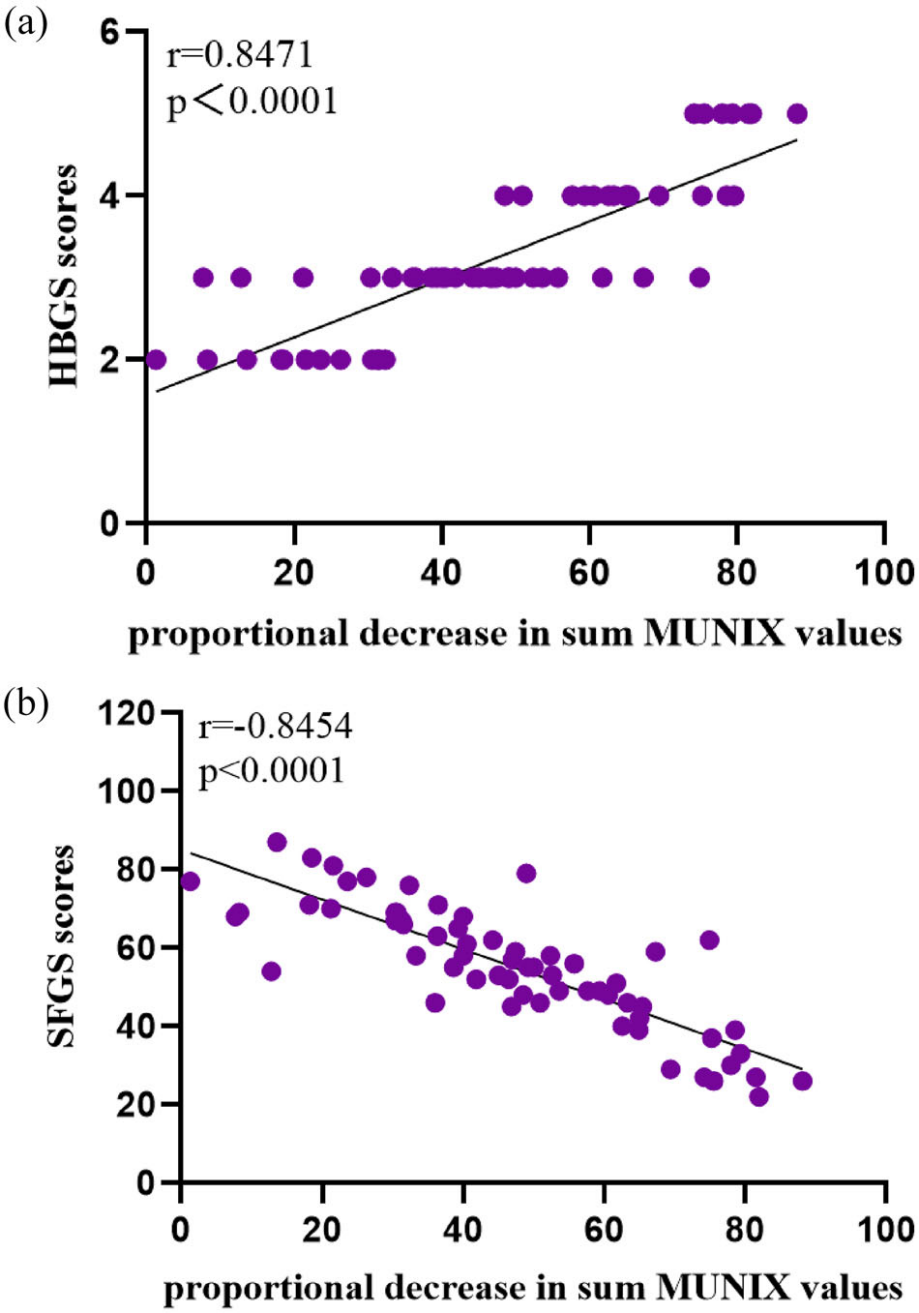
Linear regression between the proportional decrease in sum MUNIX values and (a) House–Brackmann Grading System (HBGS) scores, Spearman correlation coefficient 0.8471 (*p* < .0001), (b) Sunnybrook Facial Grading System (SFGS) scores, *R* ^2^ = 0.7146, Pearson correlation coefficient −0.8454 (*p* < .0001). Proportional decrease in sum MUNIX values (%) = (healthy side SUM MUNIX‐affected side SUM MUNIX)/healthy side SUM MUNIX. HBGS scores: six levels (1–6), 6 is complete paralysis, 1 is normal function. SFGS scores: 0–100, 0 is complete paralysis, 100 is normal function. (*n* = 64).

In Bell's palsy patients, the proportional decrease in sum MUNIX values is positively correlated with HBGS scores (*r* = 0.8472, *p* < .0001) (Figure 4a). The proportional decrease in sum MUNIX values was negatively correlated with the SFGS scores (*R* ^2^ = 0.7146, *r* = −0.8454, *p* < .0001) (Figure 4b). A low proportional decrease in sum MUNIX values indicates minor motor impairment discrepancies between the affected and unaffected sides, this is associated with low HBGS scores and high SFGS scores.

### 3.5 Changes following treatment

**FIGURE 5:**
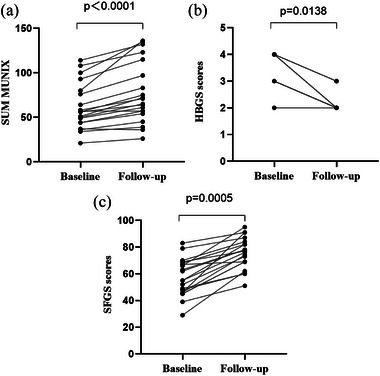
(a) Scatterplots demonstrating changes in MUNIX sum scores between repeat appointments in Bell's palsy patients receiving standardized treatment. (b) Scatterplots demonstrating changes in HBGS scores between repeat appointments in Bell's palsy patients receiving standardized treatment. (c) Scatterplots demonstrating changes in SFGS scores between repeat appointments in Bell's palsy patients receiving standardized treatment. (*n* = 20).

The MUNIX values of 20 of the 64 enrolled patients before and after treatment were tested for conformity with normal distribution by the Shapiro−Wilk test. Short‐term improvement was seen in SUM MUNIX scores comparing studies performed before and 1‐month following standardized treatment (mean value 61.4 to 77.15 *p*<.0001) (Figure 5a). Meanwhile, improvements were seen in HBGS scores (mean value 3.15 to 2.15 *p* = .0138<.05) (Figure 5b) and SFGS scores (mean value 56.8 to 75.4 *p* = .0005<.05) (Figure 5c).

### 3.6 Correlations between baseline SUM MUSIX and follow‐up clinical assessments

**FIGURE 6:**
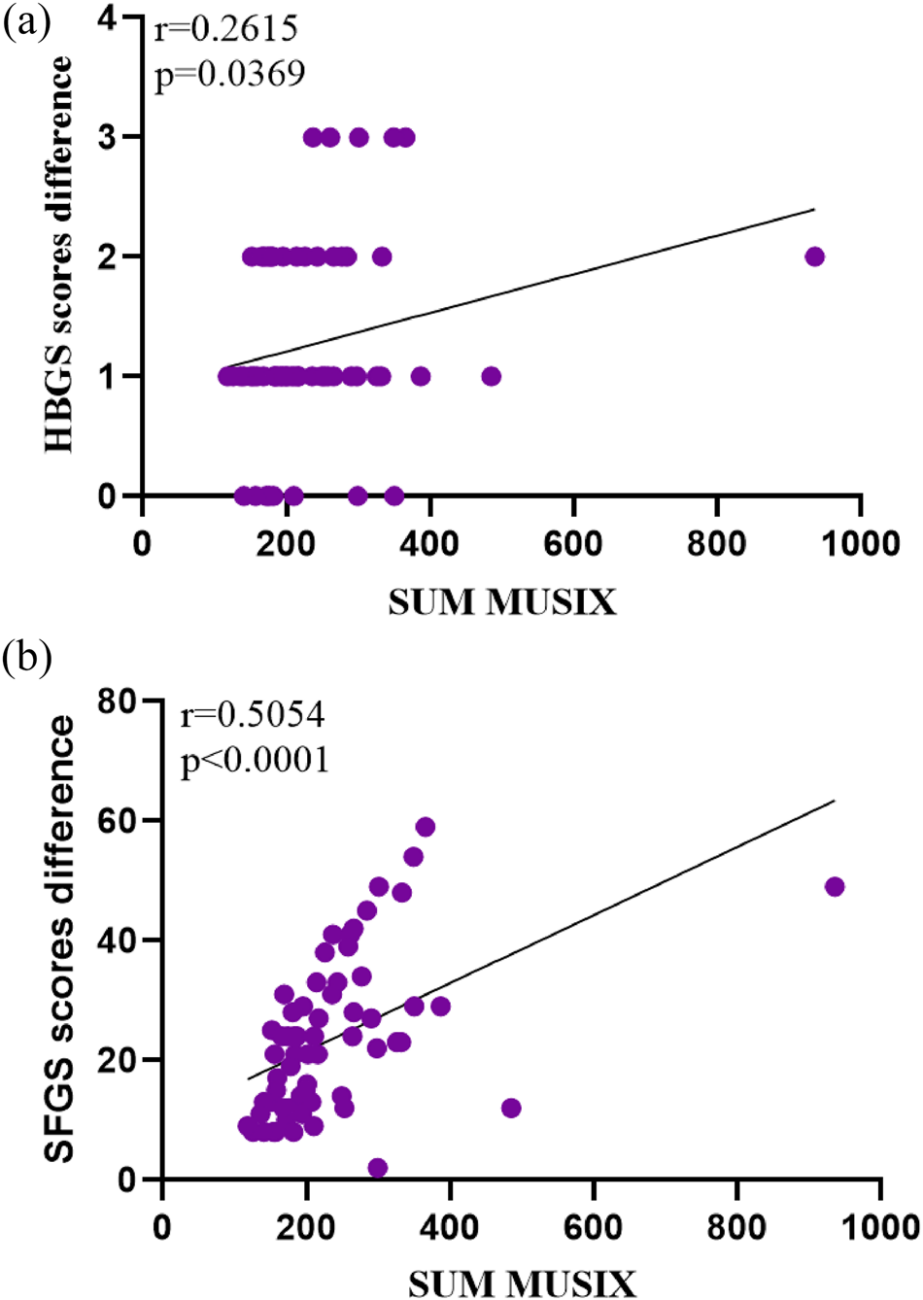
Linear regression between baseline SUM MUSIX and (a) HBGS scores difference, Spearman correlation coefficient 0.2615 (*p*=.0369<.05), (b) Sunnybrook Facial Grading System (SFGS) scores, *R* ^2^ = 0.2554, Pearson correlation coefficient 0.5054 (*p* < .0001). HBGS scores difference = baseline HBGS scores — follow‐up HBGS scores. SFGS scores difference = follow‐up SFGS scores — baseline SFGS scores. HBGS scores: six levels (1—6), 6 is complete paralysis, 1 is normal function. SFGS scores: 0–100, 0 is complete paralysis, 100 is normal function. (*n* = 64).

In Bell's palsy patients, the baseline SUM MUSIX(*n* = 64)is positively correlated with HBGS scores difference after 1 month following standardized treatment (*r* = 0.2615, *p* = .0369<.05) (Figure 6a). The baseline SUM MUSIX(*n* = 64)is positively correlated with SFGS scores difference after 1 month following standardized treatment (*R* ^2^ = 0.2554, *r* = 0.5054, *p* < .0001) (Figure 6b). Early baseline SUM MUSIX in patients with Bell's palsy can be somewhat indicative of a patient's clinical prognosis, with the magnitude of the value positively correlating with the degree of clinical recovery at 1 month.

## 4 DISCUSSION

The results of this study showed a significant decrease in MUNIX values for individual muscles and MUNIX sum values on the affected side compared with the healthy side in patients with Bell's palsy. Repeatability testing after 1 month suggested that this is a dependable method for measuring changes in motor unit function over time. The application of MUNIX sum values increased test–retest reliability compared with the use of MUNIX alone, which relies on a single muscle. Reduced MUNIX sum values were associated with clinical assessment scales in patients with Bell's palsy, demonstrating a significant correlation between the MUNIX sum values and the degree of facial nerve damage. Patients with Bell's palsy who had significantly decreased MUNIX values exhibited more severe facial muscle paralysis and disability, reflecting the decreased number of functional motor units. Short‐term improvement was observed in MUNIX sum scores when comparing results from before and 1 month following standardized treatment. In comparison, routine electrophysiological tests, such as ENoG, exhibit numerous limitations with regard to the assessment of changes in patient conditions (Adams & Katirji, 2022; Baugh et al., 2013b). Therefore, objective electrophysiological evidence was not previously available for assessing the efficacy of patient treatment. MUNIX can be useful in following up changes in patients with Bell's palsy and evaluating the efficacy of patient treatments, and is thus expected to provide guidance for clinical interventions as future studies are refined.

Nandedkar et al. have shown that as MUSIX reflects nerve regeneration, its evaluation can describe the process of reinnervation in neuromuscular diseases (Nandedkar et al., 2010). As the degree of symptom progression in neuromuscular diseases depends on the amount of axonal loss and effective reinnervation, the MUSIX value may reveal the changes in the natural course of neuromuscular diseases and can reflect important information, such as the response of the disease to therapeutic methods as well as disease prognosis. According to our results, the total MUSIX values in the early stages of Bell's palsy are somewhat indicative of patient prognosis and positively correlated with the degree of clinical recovery at 1 month. The early increase in MUSIX also suggests early reinnervation of the facial nerve in patients with idiopathic facial nerve palsy, indicating a positive effect of compensatory reinnervation on disease recovery. Although additional studies are required, these findings suggest that MUSIX may provide important guidance in uncovering the pathogenesis of idiopathic facial nerve palsy and the mechanism of disease recovery.

Previous studies have demonstrated the value of MUNIX as a potential electrophysiological biomarker for various neurological disorders (Boulay et al., 2021; Escorcio‐Bezerra et al., 2016; Fatehi et al., 2017; Furtula et al., 2013). MUNIX has been used in multiple clinical studies; in particular, it serves as a reliable electrophysiological biomarker to track amyotrophic lateral sclerosis (ALS) disease progression as well as presymptomatic ALS (Escorcio‐Bezerra et al., 2018; Fukada et al., 2016; Neuwirth et al., 2015) and as a sensitive indicator of motor unit loss in adult‐type or late‐onset spinal muscular atrophy in children (Querin et al., 2018; Verma et al., 2020). Moreover, as MUNIX values are closely related to neurological function scores, it can be used to monitor treatment effectiveness in patients with chronic inflammatory demyelinating polyneuropathy (Delmont et al., 2016; Lawley et al., 2019).

In the present study, we validated the use of MUNIX for clinical application in Bell's palsy. Our results suggest the feasibility of MUNIX for quantitatively evaluating early facial nerve dysfunction and the severity of facial nerve palsy in patients with Bell's palsy, supporting its use as a clinical tool for the quantitative and reliable evaluation of facial nerve function. In particular, the three muscles selected for evaluation were easy to assess and were considered most likely to be affected by facial nerve palsy (Heckmann et al., 2019). The MUNIX test was repeated to demonstrate the reliability of the readings from the three facial muscles. In addition, the MUNIX method used in this study is well tolerated and not time‐consuming, usually requiring approximately 20 min.

Moreover, we demonstrated that the assessment of MUNIX values for Bell's nerve palsy within 7 days of onset is meaningful, providing objective electrophysiological evidence for patients with a confirmed diagnosis of Bell's palsy and showing an inverse correlation between total MUNIX values and disease severity. Quantification of the severity of Bell's palsy in patients at an early disease stage is expected to guide the establishment of precise individualized treatments and reduce the incidence of poor prognoses. Accordingly, it is recommended that the MUNIX test be performed in patients with early Bell's palsy to assess disease severity, with regular dynamic reviews to assess recovery and provide timely guidance on medication and treatment adjustments.

This study has several limitations. Owing to the striking clinical manifestations of Bell's palsy, it is not possible for clinicians performing the MUNIX test to be blinded to the affected side. In addition, this study focused on the early assessment of patients with Bell's palsy, whereas a relatively small number of patients were evaluated to explore the value of MUNIX for follow‐up. We will confirm the utility of MUNIX for follow‐up and prognosis evaluation in Bell's palsy in future studies. Further evaluation is also warranted with regard to increased follow‐up duration and the combination of MUNIX and routine electromyography methods (e.g., EnoG, BR, and nEMG). Finally, the reliability of MUNIX results may be influenced not only by the cooperation of the person being tested, but also by technical aspects, such as the accuracy of electrode positioning. Therefore, before the test, a professional electromyographer should instruct the patient regarding the standard test movements and verify cooperation, repeating the test as necessary to obtain quality data. It is also important to set standard electrode positioning and maintain recording patch fit and reinforcement (Neuwirth et al., 2017).

## 5 CONCLUSION

MUNIX is a new, noninvasive, and simple method for performing neurophysiological testing. It can be used to complement routine electrophysiological tests to quantitatively and reliably assess the function of the facial nerve in patients with early Bell's palsy, determine disease severity and prognosis, and ultimately guide clinical treatments. MUNIX is thus a useful electrophysiological biomarker for early evaluation in Bell's palsy and holds promise for application in other disorders.

## AUTHOR CONTRIBUTIONS

**Mengjie Chen**: Conceptualization; data curation; formal analysis; investigation; methodology; writing—original draft; writing—review and editing. **Mingxia Zhu**: Formal analysis; investigation; methodology. **Xiuli Li**: Data curation; investigation. **Jingtao Pi**: Data curation. **Xinhong Feng**: Conceptualization; data curation; formal analysis; funding acquisition; investigation; methodology; project administration; resources; software; supervision; validation; visualization; writing—original draft; writing—review and editing.

## CONFLICT OF INTEREST STATEMENT

None of the authors have any conflict of interest to disclose.

### PEER REVIEW

The peer review history for this article is available at https://publons.com/publon/10.1002/brb3.3632

## Data Availability

The data that support the findings of this study are available on request from the corresponding author. The data are not publicly available due to privacy or ethical restrictions.

## References

[brb33632-bib-0001] Adams, R. , & Katirji, B. (2022). Neuromuscular ultrasound findings in acute conduction block neuropathy. Muscle & Nerve, 66, S32–S32.

[brb33632-bib-0002] Azuma, T. , Nakamura, K. , Takahashi, M. , Miyoshi, H. , Toda, N. , Iwasaki, H. , Fuchigami, T. , Sato, G. , Kitamura, Y. , Abe, K. , & Takeda, N. (2020). Electroneurography cannot predict when facial synkinesis develops in patients with facial palsy. Journal of Medical Investigation, 67(1.2), 87–89. 10.2152/jmi.67.87 32378624

[brb33632-bib-0003] Azuma, T. , Nakamura, K. , Takahashi, M. , Miyoshi, H. , Toda, N. , Iwasaki, H. , & Takeda, N. (2018). Electroneurography in the acute stage of facial palsy as a predictive factor for the development of facial synkinesis sequela. Auris, Nasus, Larynx, 45(4), 728–731. 10.1016/j.anl.2017.09.016 28966005

[brb33632-bib-0004] Baugh, R. F. , Basura, G. J. , Ishii, L. E. , Schwartz, S. R. , Drumheller, C. M. , Burkholder, R. , Deckard, N. A. , Dawson, C. , Driscoll, C. , Gillespie, M. B. , Gurgel, R. K. , Halperin, J. , Khalid, A. N. , Kumar, K. A. , Micco, A. , Munsell, D. , Rosenbaum, S. , & Vaughan, W. (2013a). Clinical practice guideline: Bell's palsy. Otolaryngology ‐ Head and Neck Surgery, 149(3 Suppl), S1–27. 10.1177/0194599813505967 24189771

[brb33632-bib-0005] Baugh, R. F. , Basura, G. J. , Ishii, L. E. , Schwartz, S. R. , Drumheller, C. M. , Burkholder, R. , Deckard, N. A. , Dawson, C. , Driscoll, C. , Gillespie, M. B. , Gurgel, R. K. , Halperin, J. , Khalid, A. N. , Kumar, K. A. , Micco, A. , Munsell, D. , Rosenbaum, S. , & Vaughan, W. (2013b). Clinical practice guideline: Bell's palsy executive summary. Otolaryngology ‐ Head and Neck Surgery, 149(5), 656–663. 10.1177/0194599813506835 24190889

[brb33632-bib-0006] Boulay, C. , Delmont, E. , Audic, F. , Chabrol, B. , & Attarian, S. (2021). Motor unit number index: A potential electrophysiological biomarker for pediatric spinal muscular atrophy. Muscle & Nerve, 64(4), 445–453. 10.1002/mus.27372 34255873

[brb33632-bib-0007] Delmont, E. , Benvenutto, A. , Grimaldi, S. , Duprat, L. , Philibert, M. , Pouget, J. , Grapperon, A. M. , Salort‐Campana, E. , Sévy, A. , Verschueren, A. , & Attarian, S. (2016). Motor unit number index (MUNIX): Is it relevant in chronic inflammatory demyelinating polyradiculoneuropathy (CIDP)? Clinical Neurophysiology, 127(3), 1891–1894. 10.1016/j.clinph.2015.12.002 26750580

[brb33632-bib-0008] Escorcio‐Bezerra, M. L. , Abrahao, A. , de Castro, I. , Chieia, M. A. T. , De Azevedo, L. A. , Pinheiro, D. S. , de Oliveira Braga, N. I. , de Oliveira, A. S. B. , & Manzano, G. M. (2016). MUNIX: Reproducibility and clinical correlations in amyotrophic lateral sclerosis. Clinical Neurophysiology, 127(9), 2979–2984. 10.1016/j.clinph.2016.06.011 27458836

[brb33632-bib-0009] Escorcio‐Bezerra, M. L. , Abrahao, A. , Nunes, K. F. , de Oliveira Braga, N. I. , Oliveira, A. S. B. , Zinman, L. , & Manzano, G. M. (2018). Motor unit number index and neurophysiological index as candidate biomarkers of presymptomatic motor neuron loss in amyotrophic lateral sclerosis. Muscle & Nerve, 58(2), 204–212. 10.1002/mus.26087 29381812

[brb33632-bib-0010] Eviston, T. J. , Croxson, G. R. , Kennedy, P. G. E. , Hadlock, T. , & Krishnan, A. V. (2015). Bell's palsy: Aetiology, clinical features and multidisciplinary care. Journal of Neurology, Neurosurgery, and Psychiatry, 86(12), 1356–1361. 10.1136/jnnp-2014-309563 25857657

[brb33632-bib-0011] Fatehi, F. , Delmont, E. , Grapperon, A. M. , Salort‐Campana, E. , Sévy, A. , Verschueren, A. , Boucraut, J. , & Attarian, S. (2017). Motor unit number index (MUNIX) in patients with anti‐MAG neuropathy. Clinical Neurophysiology, 128(7), 1264–1269. 10.1016/j.clinph.2017.04.022 28545015

[brb33632-bib-0012] Fukada, K. , Matsui, T. , Furuta, M. , Hirozawa, D. , Matsui, M. , Kajiyama, Y. , Shimizu, M. , Kinoshita, M. , Mochizuki, H. , Sawada, J. I. , & Hazama, T. (2016). The motor unit number index of subclinical abnormality in amyotrophic lateral sclerosis. Journal of Clinical Neurophysiology, 33(6), 564–568. 10.1097/WNP.0000000000000296 27295331

[brb33632-bib-0013] Furtula, J. , Johnsen, B. , Christensen, P. B. , Pugdahl, K. , Bisgaard, C. , Christensen, M. K. , Arentsen, J. , Frydenberg, M. , & Fuglsang‐Frederiksen, A. (2013). MUNIX and incremental stimulation MUNE in ALS patients and control subjects. Clinical Neurophysiology, 124(3), 610–618. 10.1016/j.clinph.2012.08.023 23040293

[brb33632-bib-0014] George, E. , Richie, M. B. , & Glastonbury, C. M. (2020). Facial nerve palsy: Clinical practice and cognitive errors. American Journal of Medicine, 133(9), 1039–1044. 10.1016/j.amjmed.2020.04.023 32445717

[brb33632-bib-0015] Granger, C. V. (1976). Prognosis in Bell's palsy. Archives of Physical Medicine and Rehabilitation, 57(1), 33–35. 1247374

[brb33632-bib-0016] Guntinas‐Lichius, O. , Volk, G. F. , Olsen, K. D. , Mäkitie, A. A. , Silver, C. E. , Zafereo, M. E. , Rinaldo, A. , Randolph, G. W. , Simo, R. , Shaha, A. R. , Vander Poorten, V. , & Ferlito, A. (2020). Facial nerve electrodiagnostics for patients with facial palsy: A clinical practice guideline. European Archives of Oto‐Rhino‐Laryngology, 277(7), 1855–1874. 10.1007/s00405-020-05949-1 32270328 PMC7286870

[brb33632-bib-0017] Heckmann, J. G. , Urban, P. P. , Pitz, S. , Guntinas‐Lichius, O. , & Gágyor, I. (2019). The diagnosis and treatment of idiopathic facial paresis (Bell's palsy). Deutsches Ärzteblatt International, 116(41), 692–702. 10.3238/arztebl.2019.0692 31709978 PMC6865187

[brb33632-bib-0018] House, J. W. , & Brackmann, D. E. (1985). Facial nerve grading system. Otolaryngology ‐ Head and Neck Surgery, 93(2), 146–147. 10.1177/019459988509300202 3921901

[brb33632-bib-0019] Kennelly, K. D. (2019). Clinical neurophysiology of cranial nerve disorders. Handbook of Clinical Neurology, 161, 327–342. 10.1016/B978-0-444-64142-7.00058-8 31307611

[brb33632-bib-0020] Koo, T. K. , & Li, M. Y. (2016). A guideline of selecting and reporting intraclass correlation coefficients for reliability research. Journal of Chiropractic Medicine, 15(2), 155–163. 10.1016/j.jcm.2016.02.012 27330520 PMC4913118

[brb33632-bib-0021] Lawley, A. , Seri, S. , & Rajabally, Y. A. (2019). Motor unit number index (MUNIX) in chronic inflammatory demyelinating polyneuropathy: A potential role in monitoring response to intravenous immunoglobulins. Clinical Neurophysiology, 130(10), 1743–1749. 10.1016/j.clinph.2019.06.231 31400577

[brb33632-bib-0022] Nandedkar, S. D. , Barkhaus, P. E. , & Stålberg, E. V. (2010). Motor unit number index (MUNIX): Principle, method, and findings in healthy subjects and in patients with motor neuron disease. Muscle & Nerve, 42(5), 798–807. 10.1002/mus.21824 20976783

[brb33632-bib-0023] Nandedkar, S. D. , Barkhaus, P. E. , Stålberg, E. V. , Neuwirth, C. , & Weber, M. (2018). Motor unit number index: Guidelines for recording signals and their analysis. Muscle & Nerve, 58(3), 374–380. 10.1002/mus.26099 29427557

[brb33632-bib-0024] Nandedkar, S. D. , Nandedkar, D. S. , Barkhaus, P. E. , & Stalberg, E. V. (2004). Motor unit number index (MUNIX). IEEE Transactions on Bio‐Medical Engineering, 51(12), 2209–2211. 10.1109/TBME.2004.834281 15605872

[brb33632-bib-0025] Neely, J. G. , Cherian, N. G. , Dickerson, C. B. , & Nedzelski, J. M. (2010). Sunnybrook facial grading system: Reliability and criteria for grading. Laryngoscope, 120(5), 1038–1045. 10.1002/lary.20868 20422701

[brb33632-bib-0026] Neuwirth, C. , Barkhaus, P. E. , Burkhardt, C. , Castro, J. , Czell, D. , de Carvalho, M. , Nandedkar, S. , Stålberg, E. , & Weber, M. (2015). Tracking motor neuron loss in a set of six muscles in amyotrophic lateral sclerosis using the Motor Unit Number Index (MUNIX): A 15‐month longitudinal multicentre trial. Journal of Neurology, Neurosurgery, and Psychiatry, 86(11), 1172–1179. 10.1136/jnnp-2015-310509 25935892

[brb33632-bib-0027] Neuwirth, C. , Barkhaus, P. E. , Burkhardt, C. , Castro, J. , Czell, D. , de Carvalho, M. , Nandedkar, S. , Stålberg, E. , & Weber, M. (2017). Motor Unit Number Index (MUNIX) detects motor neuron loss in pre‐symptomatic muscles in Amyotrophic Lateral Sclerosis. Clinical Neurophysiology, 128(3),495–500. 10.1016/j.clinph.2016.11.026 28043769

[brb33632-bib-0028] Querin, G. , Lenglet, T. , Debs, R. , Stojkovic, T. , Behin, A. , Salachas, F. , Le Forestier, N. , Amador, M. D. M. , Lacomblez, L. , Meininger, V. , Bruneteau, G. , Laforêt, P. , Blancho, S. , Marchand‐Pauvert, V. , Bede, P. , Hogrel, J. Y. , & Pradat, P. F. (2018). The motor unit number index (MUNIX) profile of patients with adult spinal muscular atrophy. Clinical Neurophysiology, 129(11), 2333–2340. 10.1016/j.clinph.2018.08.025 30248623

[brb33632-bib-0029] Remenschneider, A. K. , Michalak, S. , Kozin, E. D. , Barber, S. , de Venecia, R. K. , Hadlock, T. A. , & Jung, D. H. (2017). Is serial electroneuronography indicated following temporal bone trauma? Otology & Neurotology, 38(4), 572–576. 10.1097/MAO.0000000000001337 28114180

[brb33632-bib-0030] Ross, B. R. , Fradet, G. , & Nedzelski, J. M. (1994). Development of a sensitive clinical facial grading system. European Archives of Oto‐Rhino‐Laryngology, S180–S181. 10.1007/978-3-642-85090-5_63 10774344

[brb33632-bib-0031] Tiemstra, J. D. , & Khatkhate, N. (2007). Bell's palsy: Diagnosis and management. American Family Physician, 76(7), 997–1002. 17956069

[brb33632-bib-0032] Urban, E. , Volk, G. F. , Geißler, K. , Thielker, J. , Dittberner, A. , Klingner, C. , Witte, O. W. , & Guntinas‐Lichius, O. (2020). Prognostic factors for the outcome of Bells' palsy: A cohort register‐based study. Clinical Otolaryngology, 45(5), 754–761. 10.1111/coa.13571 32395899

[brb33632-bib-0033] Verma, S. , Forte, J. , Ritchey, M. , & Shah, D. (2020). Motor unit number index in children with later‐onset spinal muscular atrophy. Muscle & Nerve, 62(5), 633–637. 10.1002/mus.26909 32369629

[brb33632-bib-0034] Yen, T. L. , Driscoll, C. L. W. , & Lalwani, A. K. (2003). Significance of House–Brackmann facial nerve grading global score in the setting of differential facial nerve function. Otology & Neurotology, 24(1), 118–122. 10.1097/00129492-200301000-00023 12544040

